# Supplementary material for: Cryo-EM structures reveal the PP2A–B55α and Eya3 interaction that can be disrupted by a peptide inhibitor
Source: J Biol Chem. 2025 May 23;301(7):110287. doi: 10.1016/j.jbc.2025.110287 (PMC12246617; doi:10.1016/j.jbc.2025.110287)
Supplement: Supporting Information [file mmc1.pdf]

Fig. S1

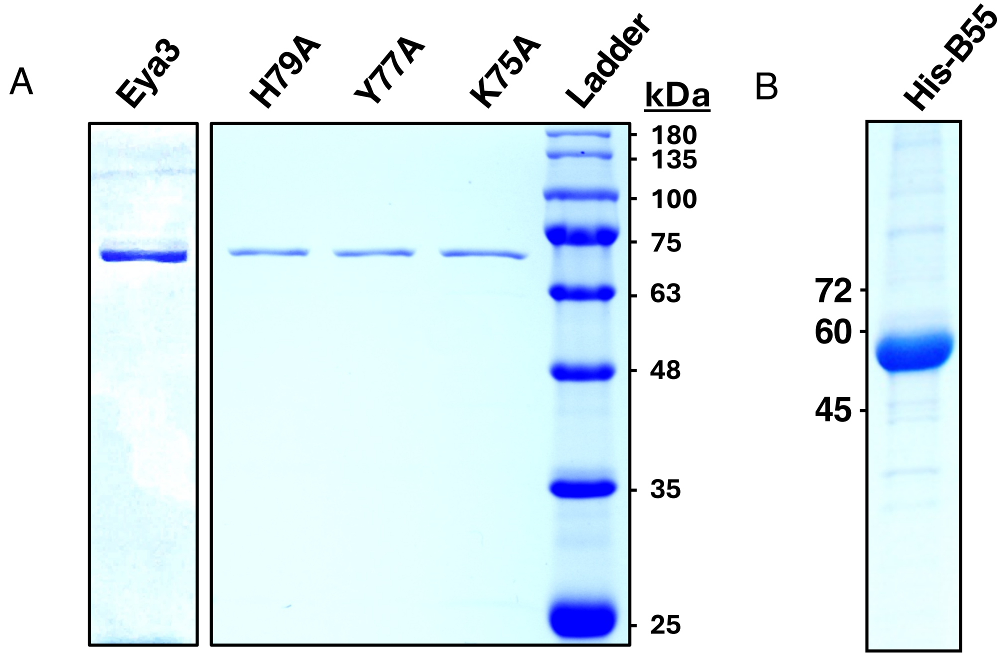

Fig. S1. Eya3 and His-B55 purification. A) Eya3 WT and mutants purified from *E. coli* and B) His-B55 purified from insect cell are shown on Coomassie stained gels.

Fig. S2

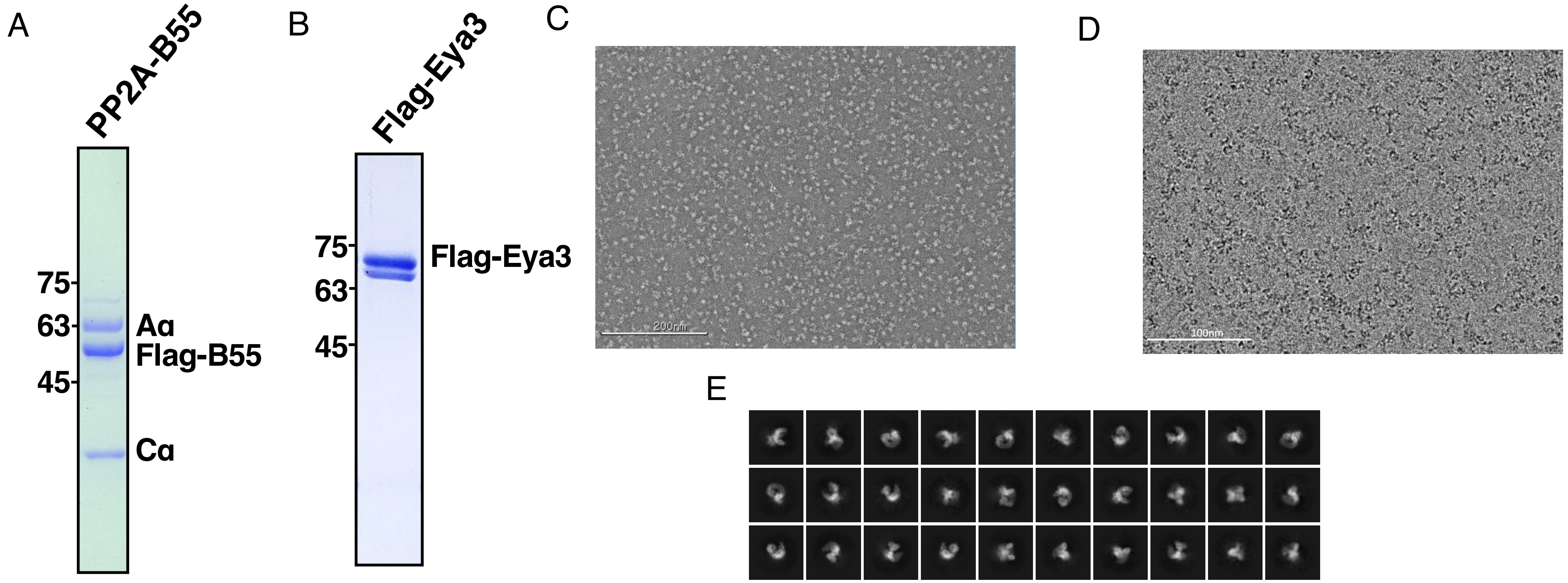

Fig. S2. Structural determination of the PP2A-B55 + Eya3 complex. A) PP2A-B55 complex purified from FreeStyle HEK293 cells shown on Coomassie stained gel. B) Flag-tagged human Eya3 purified from FreeStyle HEK293 cells shown on Coomassie stained gel. C) A representative negative stain image of the PP2A-B55 + Eya3 complex. D) A representative cryo image of the PP2A-B55 + Eya3 complex. E) 2D classification of the cryo-EM data.

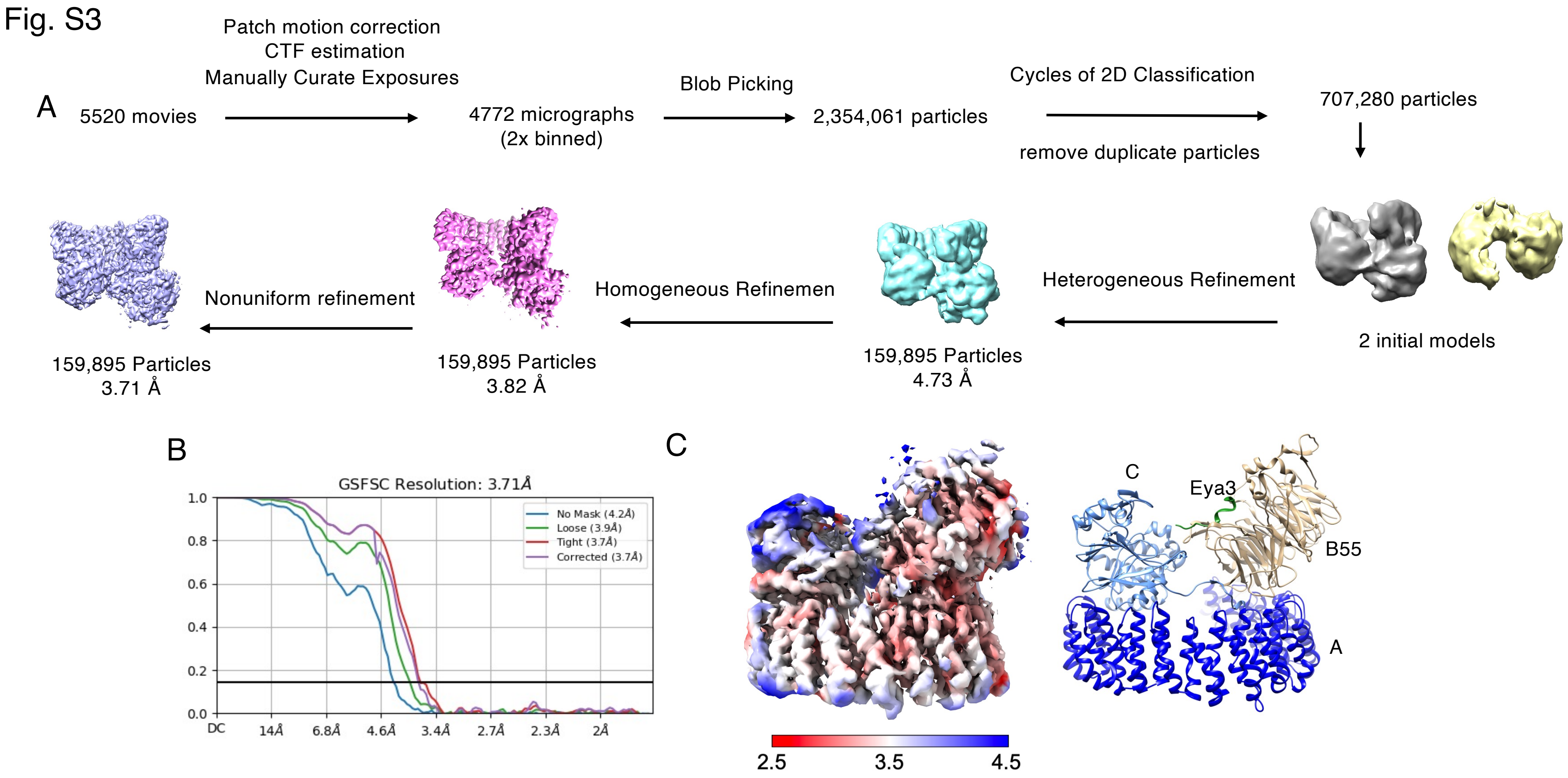

Fig. S3. A) Workflow of the structural determination process. B) Gold-Standard FSC curves show the overall resolution of the structure. C) Cryo-EM map of the structure colored by local resolutions. A ribbon diagram of the structure is also shown to illustrate the orientation of the local resolution map.

Fig. S4

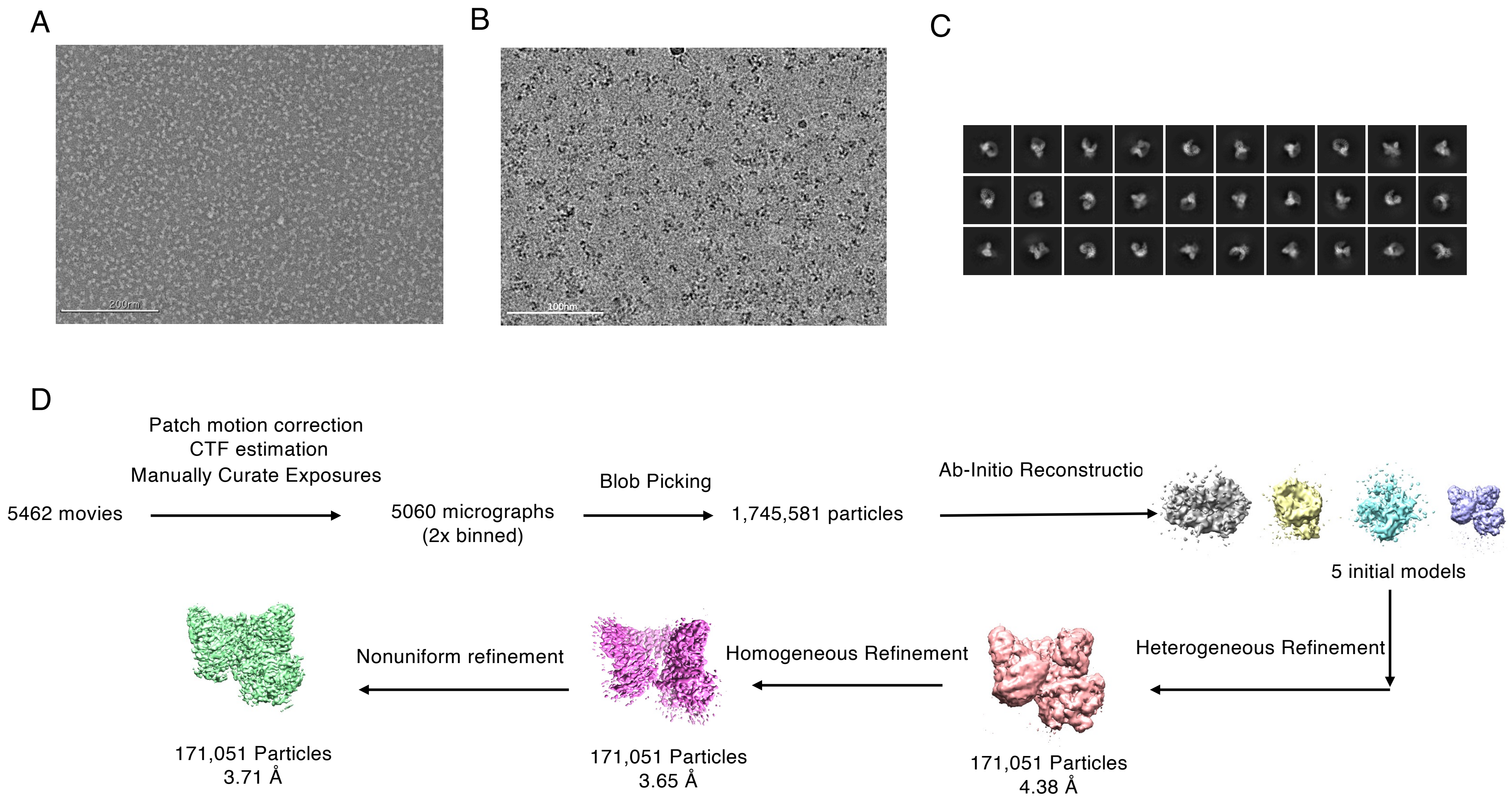
A
B
C
200nm
100nm
D
Patch motion correction
CTF estimation
Manually Curate Exposures
5462 movies
5060 micrographs
(2x binned)
Blob Picking
1,745,581 particles
Ab-Initio Reconstructio
5 initial models
Heterogeneous Refinement
171,051 Particles
4.38 Å
Homogeneous Refinement
171,051 Particles
3.65 Å
Nonuniform refinement
171,051 Particles
3.71 Å

Fig. S4

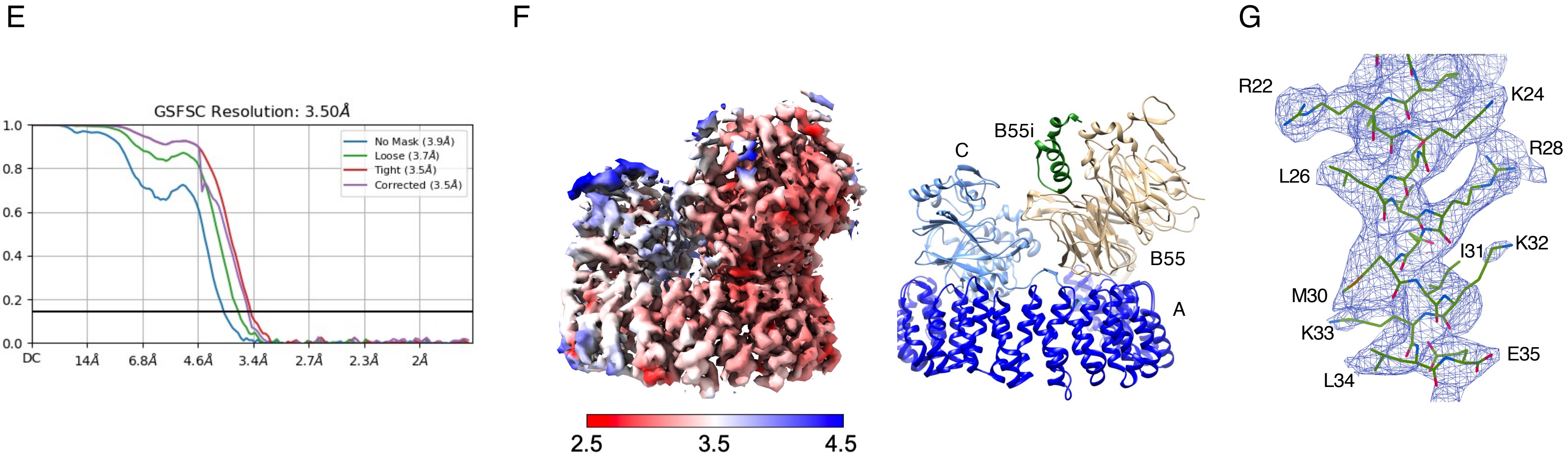

Fig. S4. Structural determination of the PP2A-B55 + B55i complex. A) A representative negative stain image of the PP2A-B55 + B55i complex. B) A representative cryo image of the PP2A-B55 + B55i complex. C) 2D classification of the cryo-EM data. D) Workflow of the structural determination process. E) Gold-Standard FSC curves show the overall resolution of the structure. F) Cryo-EM map of the structure colored by local resolutions. A ribbon diagram of the structure is also shown to illustrate the orientation of the local resolution map. G) Density for the C-terminal helix (residues 22-35) of B55i.

Fig. S5

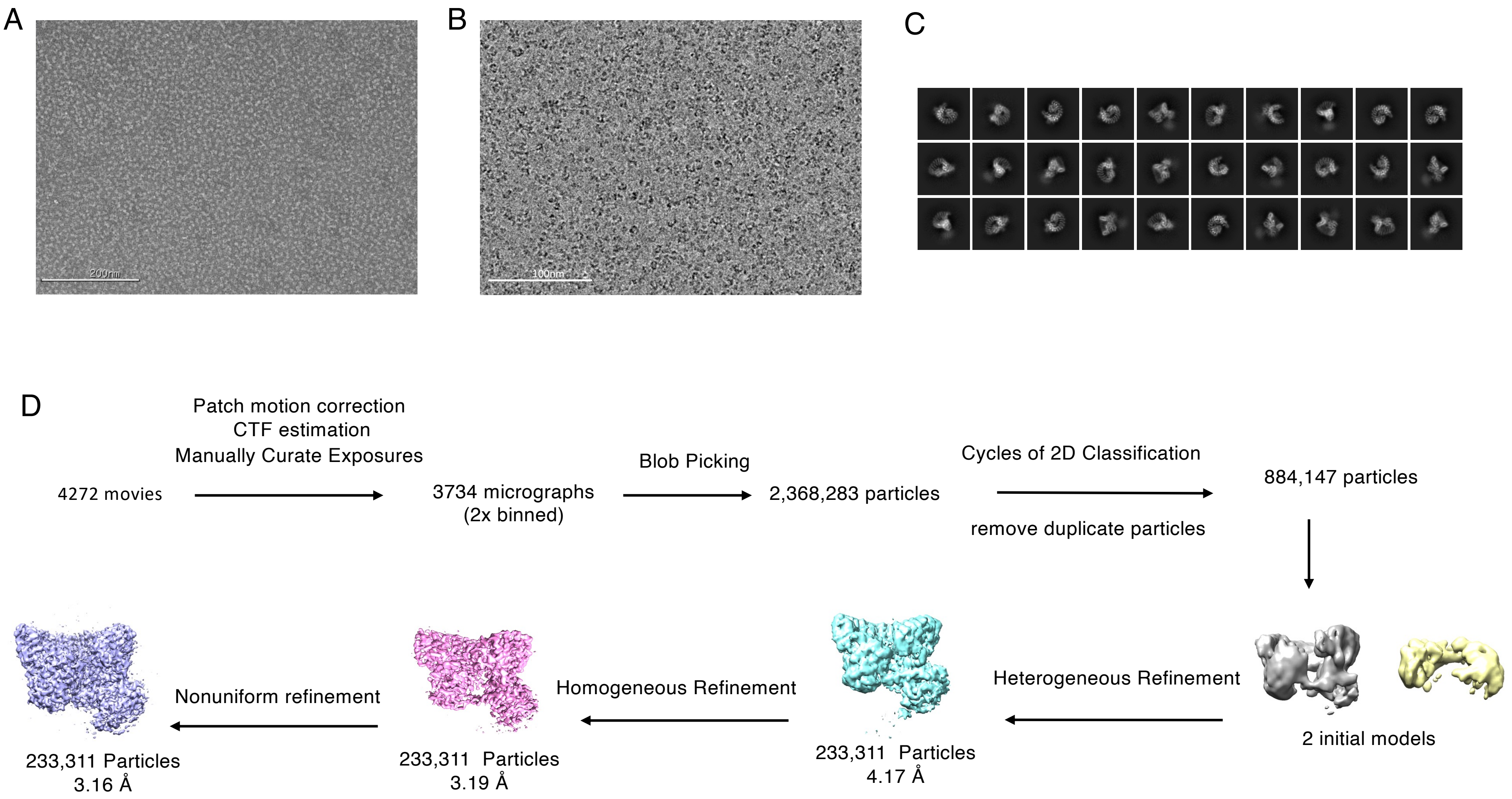

A
200nm
B
100nm
C
D
4272 movies
Patch motion correction
CTF estimation
Manually Curate Exposures
3734 micrographs
(2x binned)
Blob Picking
2,368,283 particles
Cycles of 2D Classification
remove duplicate particles
884,147 particles
2 initial models
Heterogeneous Refinement
233,311 Particles
4.17 Å
Homogeneous Refinement
233,311 Particles
3.19 Å
Nonuniform refinement
233,311 Particles
3.16 Å

Fig. S5

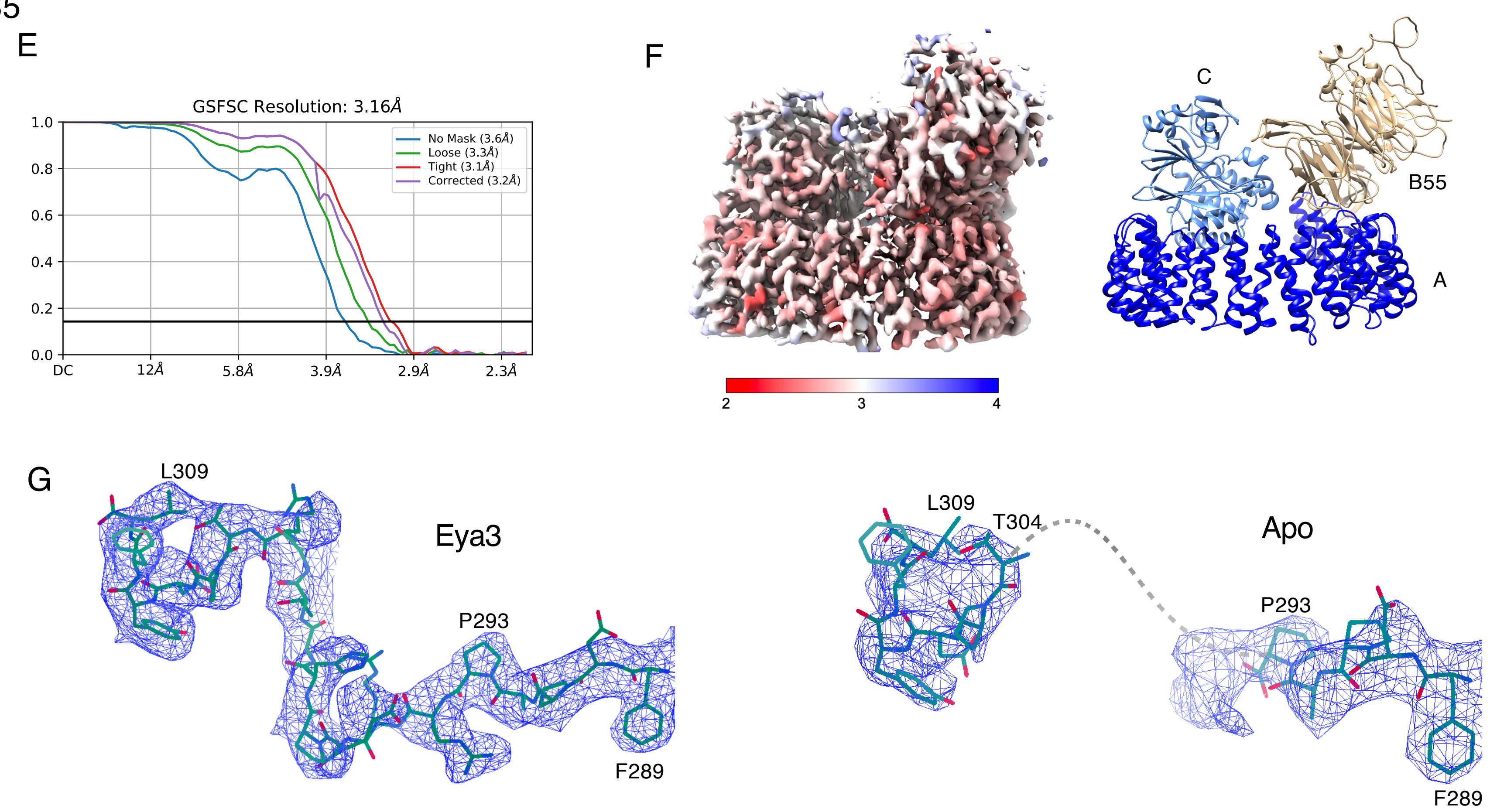

Fig. S5. Structural determination of the apo PP2A-B55 complex. A) A representative negative stain image of the apo PP2A-B55 complex. B) A representative cryo image of the apo PP2A-B55 complex. C) 2D classification of the cryo-EM data. D) Workflow of the structural determination process. E) Gold-Standard FSC curves show the overall resolution of the structure. F) Cryo-EM map of the structure colored by local resolutions. A ribbon diagram of the structure is also shown to illustrate the orientation of the local resolution map. G) Density for the C-terminal tail in the PP2A-B55 + Eya3 and apo structures.

Table S1. Data statistics for structural determination and refinement.

	PP2A-B55 (EMD-48770) (PDB 9MZW)	PP2A-B55 + Eya3 (EMD-48798) (PDB 9N0Y)	PP2A-B55 + B55i (EMD-48799) (PDB 9N0Z)
**Data collection, processing**			
Magnification	81,000	105,000	105,000
Voltage (Kv)	300	300	300
Exposure dose ((e-/Å2))	54	50.02	50.02
Defocus range (µm)	−1~−2.5	−0.6 ~ −2.0	−0.6 ~ −2.0
Pixel size (Å)	0.535	0.855	0.855
Movies collected	4272	5520	546200
Symmetry imposed	C1	C1	C1
Initial particle images (no.)	2,368,283	2,354,061	1,745,581
Final particle images (no.)	233,311	159,895	171,051
Map resolution (Å)	3.16	3.71	3.5
FSC threshold	0.143	0.143	0.143
Map resolution range	2.3-4.2	2.5-5.5	2.5-4.9
**Refinement**			
Initial model used	8SO0	8SO0	8SO0
model composition			
Chains	3	4	4
Atoms	10479	10723	13248
Residues	1312	1342	1355
R.m.s. deviations			
Bonds (Å)	0.005	0.005	0.007
Bond angles (º)	1.048	1.065	1.088
**Validation**			
MolProbity score	1.77	1.99	2.06
Clash score	8.93	8.15	9.80
Rotamer outliers (%)	0.00	0.00	0.00
Ramachandran plot			
Favored (%)	95.77	90.01	90.19
Allowed (%)	4.23	9.99	9.81
Disallowed (%)	0.00	0.00	0.00
